# Cost-effectiveness of a Dengue Vector Control Intervention in Colombia

**DOI:** 10.4269/ajtmh.20-0669

**Published:** 2022-04-18

**Authors:** Alejandra Taborda, Cindy Chamorro, Juliana Quintero, Gabriel Carrasquilla, Darío Londoño

**Affiliations:** Fundación Santa Fe de Bogotá, Bogota D.C., Colombia

## Abstract

Dengue is a public health problem in Colombia and in the municipality of Girardot, an area of high risk for dengue transmission. We present the results of an economic evaluation from the societal perspective and 1-year time horizon comparing the regular control program for dengue prevention versus an intervention that comprised an environmental management strategy by covering the most *Aedes aegypti *productive breeding sites with insecticide covers, community actions, and educational activities. The effectiveness of the intervention was measured as the reduction in probability of dengue infection obtained from a community trial. Resource use was estimated from clinical records that were validated by clinical experts; unit costs were taken from national tariffs. Patient costs were obtained from a household survey. We found that the intervention generated an additional cost of USD20.9 per household and an incremental effectiveness of 0.00173 (reduction in the probability of reported dengue cases). Overall, both alternatives generate similar effectiveness, but the new intervention was associated with increasing costs. We conclude the new intervention is a potentially cost-effective option in areas where high prevalence of dengue exists.

## INTRODUCTION

Dengue is the most common arboviral disease in the world. In 2013, 58.4 million cases and almost 14,000 deaths were estimated globally, as was a disease burden of 1.14 million disability-adjusted life years (DALYs).^1^^,^^2^ In the Americas, during 2015 and 2016 there were more than 2 million cases per year. In 2017, the reported cases decreased to 581,268; in 2018, to 533,646, with only 296 deaths.^3^ However, in 2019, more than 3 million cases were reported—the highest number in the history of the Americas and more than 30% of the number of cases reported in 2015 (an epidemic year), and these values that are likely to be much higher than official data as a result of underreported cases.^4^

In Colombia, an increasing trend has been observed during the past 25 years, from 272,360 cases between 1990 and 1999, to 454,837 cases between 2000 and 2010, and to 674,043 cases between 2010 and 2016.^5^ Fifty percent of dengue cases in Colombia (1999–2010) are reported in 18 municipalities, including Girardot, which is considered to be a high-risk area for dengue transmission.^6^

Regarding the economic impact of the disease, it is estimated that, for 2010, the region of the Americas had an annual cost of USD2.1 trillion associated with dengue.^7^ In Colombia, the annual cost was estimated at USD167.8 million for 2010, USD129.9 million for 2011, and USD131.7 million for 2012. These costs represent more than 100% of the budget allocated to the national vaccination program, 0.14% of the total national budget, and 0.036% of the gross domestic product.^8^

Well-informed decisions require economic analyses that evaluate the cost-effectiveness of the different alternatives to tackle a particular disease, to develop prevention and control strategies that reduce their burden and economic impact. Although dengue disease is a major health problem in many countries, particularly in Asia and Latin America, few studies have been reported with cost-effectiveness analyses of dengue interventions.^9^^,^^27^ A study combining vector control with community participation (in which the community identified the main health problems and needs, and elaborated, implemented, and evaluated their action plans to reduce dengue disease) versus traditional control (including larval control, blanket spraying for mosquito adulticiding, and replacement of defective water tanks) reported that the first strategy was cost-effective.^10^ Other cost-effectiveness studies in dengue have evaluated different strategies, such as insecticides for vector control,^11^ or the use of insecticide-treated school uniforms in students in Thailand, which was not cost-effective for the health system.

In Colombia, an intervention for the prevention and control of dengue vector “*Aedes *free” was carried out in Girardot, with the objective of reducing dengue incidence.^12^^,^^13^ The objective of this study was to assess the cost-effectiveness of the later intervention versus the regular vector control program for the prevention of dengue in Colombia.

## METHODS

### Type of study.

This is a model-based cost-effectiveness analysis^14^ from the societal perspective that used results from a community intervention study as the main source of effectiveness. The study compared the costs and effects of the *Aedes*-free intervention versus the regular vector control program.

### Study site.

The original trial, which was used as the main source of effectiveness for this analysis, was carried out in Girardot, Colombia, a city located 134 km from Bogota, at 289 m above sea level. Girardot has an annual average temperature of 33°C and a relative humidity of 66%. The city is characterized by persistent transmission of dengue with simultaneous circulation of all dengue serotypes. Girardot has 105,085 urban inhabitants who live in 23,885 households (97% of which are urban). Between 2010 (first epidemiological week) and 2017 (33rd epidemiological week), 3,193 suspected dengue cases were reported to the surveillance system of Girardot, of which 99.6% were classified clinically as dengue; 5.8% of dengue cases were severe, and the age group with the greatest incidence of dengue was 5 to 14 years old (SIVIGILA 2005–2017).

### *Aedes*-free intervention.

The intervention, based on an eco-health approach,^15^^,^^16^ comprised the design and installation of covers impregnated with insecticide (nets treated with long-lasting insecticide [deltamethrin 50 mg/m^2^]; Vestergaard Frandsen SA, Kolding, Denmark) in large water tanks. In addition, the intervention included community actions (which consisted of the participation of community leaders to promote the implementation of the intervention in households and to disseminate information regarding the importance of addressing the vector control actions) and strengthening knowledge of dengue and other *Aedes*-borne diseases through information and communication tools (newsletters, murals in schools, posters in neighborhood stores, and flyers). During the trial, Girardot was divided into four sectors with similar sociodemographic characteristics. Each of these was divided into intervention and control areas. During the active implementation phase 3,898 insecticide-treated aluminum covers were distributed to 2,935 households (1.32 covers per household) and 1,774 round covers with elastic band were installed in 965 households (1.84 per household). The intervention was carried out between December 2015 and February 2017 in 3,900 Girardot households. Additional details of the intervention can be found in the original publication of the trial.^13^

### Regular *Aedes* control program.

The regular control program consisted of insecticide spraying, inspection and control of potential breeding sites, and community education. Specifically, the program included physical inspections of water containers to register the presence or absence of immature forms, addition of temephos in tanks, and health education to promote changes in behavior.

### Decision model and effectiveness of the interventions.

To estimate the expected outcomes and costs of the *Aedes*-free intervention, we proposed a simple model with a 1-year time horizon (therefore, it was not necessary to use a discount rate, according to the recommendations of the Colombian Agency of Health Technology Assessment^17^) that compared the probability of dengue infection between the two interventions, and the direct and indirect costs associated with each. The analysis was performed using TreeAge Pro^®^ 2017.^18^ Because we used a 1-year time horizon, we considered that a simple decision tree would allow us to capture the most relevant outcomes of the intervention (cost of the intervention, treatment of positive cases, and probability of dengue infection in the household) and thus provide useful information to decisionmakers willing to implement a similar intervention to prevent dengue infection.

Probability of dengue with and without the intervention was derived from the data by Quintero et al.^13^ According to our study, during the 18-month period there were 64 cases of dengue in the intervention area (out of a population of 23,968) and 24 cases in the control area (out of a population of 6,049). To estimate 1-year probabilities from the 18-month rate observed in our study, we used the following formula,^14^ which assumes a constant rate during the period:

p=1–exp (−rt),

where *p* is the probability, *r* the rate, and *t* is the time period.

### Estimation of resource use and unit costs.

Considering the social perspective assumed for the evaluation, both the indirect and direct costs were included. Costs were calculated in Colombian pesos and then converted to U.S. dollars using the average exchange rate for 2017 (2,951.15 Colombian pesos for USD1).^22^

#### Direct costs of the alternatives and dengue treatment.

The total costs for the tank cover materials, technical services, human resources, logistics, and the costs of the regular program were included to obtain the costs of the intervention. The cost of the regular program considered the budget allocated in Girardot for vector control, proportional to dengue cases, and in relation to all vector-borne diseases in Girardot during the study period.

The costing process for model outcomes comprised three stages. First, we reviewed dengue guidelines and protocols to identify relevant resources used in the management of a dengue case.^21^^,^^24^ Second, we reviewed records of individual health interventions and procedures in a database of the Colombian Ministry of Health to establish quantities of each resource (hospitalization, laboratory, diagnostic procedures, and medications). Third, we used the National Institute of Social Security’s 2001 tariff manual to value medical procedures, hospitalization, and laboratory tests (adjusting the reported value by a 35% factor, with a minimum of 25% and a maximum of 48%, according to national costing guidelines). The manual, although outdated, is widely used in Colombia as a basis for negotiation between insurers and providers.^17^ Unit prices for drugs were obtained from the Drug Price Information System of Colombia.^25^

#### Indirect costs and out-of-pocket expenses.

We assumed the human capital approach to estimate productivity losses associated with dengue. Between November 2015 and August 2017, 315 interviews were conducted with Girardot residents who were reported to have dengue in the surveillance system in both the intervention and control areas. The survey included questions regarding costs incurred by the family during the time of the illness (transportation, medications, private consultation, payment of the caregiver, and other) and the time they stopped working or receiving income as a result of the illness. Indirect costs were calculated for the population in productive ages (between 15 and 64 years).

### Cost-effectiveness criteria.

To establish whether an intervention is cost-effective, the incremental cost-effectiveness ratio must be compared with a threshold of cost-effectiveness or willingness to pay.^17^^,^^26^ Given that there is no explicit threshold for the effectiveness measure we used in this analysis, we used a threshold equivalent to USD19,125 for a 1% reduction in the probability of dengue infection, which corresponds to three times the Colombian per-capita gross domestic product.

### Uncertainty and sensitivity analyses.

Two types of sensitivity analyses were performed to account for uncertainty. First, a deterministic analysis was carried out that considered the minimum and maximum values of effectiveness and costs. This analysis is presented using a tornado diagram, which shows the changes in incremental cost-effectiveness according to a univariate sensitivity analysis. We also performed another deterministic sensitivity analysis and used DALYs as alternative outcome measure. For this analysis, we used the DALYs calculator of the Center for the Evaluation of Value and Risk in Health, Tufts Medical Center (https://cevr.shinyapps.io/DALYcalculation/). For this sensitivity analysis, we assumed 20 years as the age of onset of disease for both moderate and severe dengue in male and females.

The second type of analysis conducted was a probabilistic sensitivity analysis, in which several key parameters were represented as statistical distributions. A triangular distribution was used for costs and a beta distribution was used for probabilities. We performed 1,000 iterations of the model and presented results as cost-effectiveness scatterplots and acceptability curves, which show the probability that each intervention is cost-effective for different values of the threshold.

### Ethical considerations.

The study was approved by the Research Ethics Committee of Fundación Santa Fe de Bogotá. We took all necessary steps to follow best methodological practices to conduct economic evaluations in Colombia,^17^ and ensured confidentiality of patient information.

## RESULTS

We found that incidence was greater in the control area (529 per 100,000 inhabitants) than in the intervention area (371 per 100,000 inhabitants). We also observed that the incidence increased in both sectors, but it was greater in the control areas (397 per 100,000 inhabitants) than in the intervention areas (267 per 100,000 inhabitants).^13^ Table 1 shows the estimated annual costs per household used in the model, both direct and indirect.

**Table 1:** Direct and indirect annual costs per household

Costs category	Base case, USD	Minimum case, USD	Maximum case, USD	Statistical distribution
Direct costs				
Direct medical costs	113.0	82.0	125.0	Triangular
*Aedes-*free intervention costs	21.4	18.2	24.5	Triangular
Regular program costs	9.6	6.3	12.6	Triangular
Indirect costs				
Out-of-pocket expenses	93.0	82.0	105.0	Triangular
Indirect patient costs	57.0	17.3	44.6	Triangular
Indirect caregiver costs	47.0	42.0	53.0	Triangular

USD = U.S. dollar.

Model results show that the *Aedes*-free intervention is more expensive than the regular program, with an additional cost of USD20.9 per household. Nonetheless, it also reduced the probability of dengue infection, resulting in an incremental cost-effectiveness ratio of USD12,097 (Table 2). This means the cost of reducing the probability of dengue infection in 1 point, with the *Aedes*-free intervention, requires an investment of USD12.097, which could be deemed cost-effective according to the suggested threshold.

**Table 2:** Results from the cost-effectiveness analysis (base case)

Strategy	Costs, USD	Incremental costs, USD	Effectiveness	Incremental effectiveness	ICER
Regular program	11.2	–	0.00529	–	–
Regular program + *Aedes*-free intervention	32.1	20.9	0.0356	0.00173	12,097

ICER = incremental cost-effectiveness ratio; USD = U.S. dollar.

**Figure 1.**
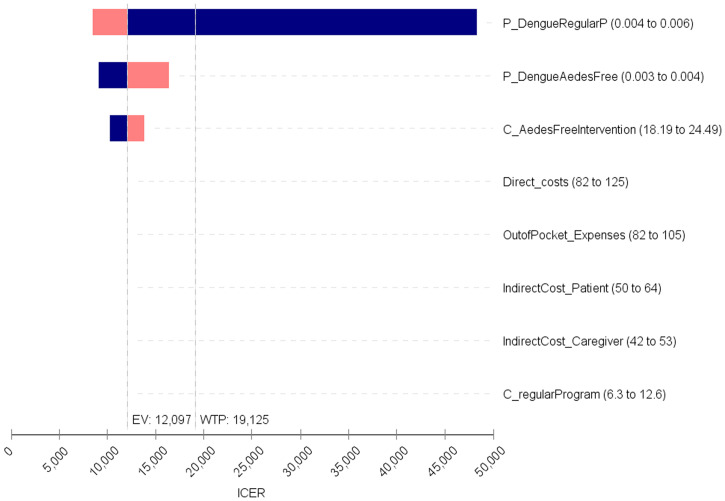
Tornado diagram: deterministic sensitivity analysis. EV = expected value; ICER = incremental cost-effectiveness ratio; WTP = willingness to pay. This figure appears in color at www.ajtmh.org.

**Figure 2.**
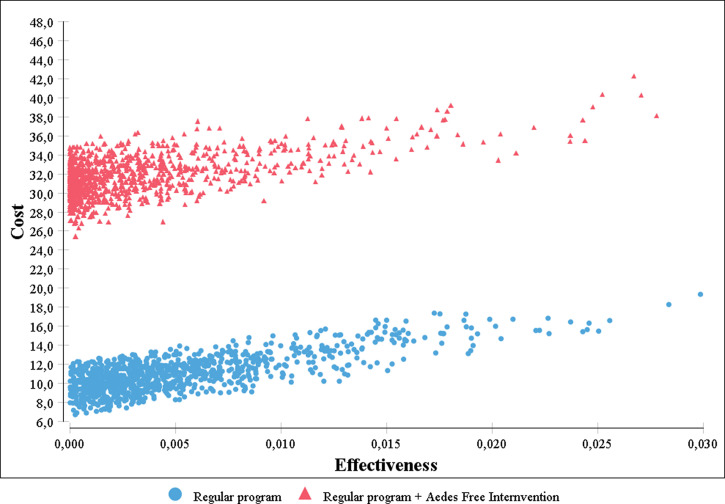
Incremental cost-effectiveness scatterplot. This figure appears in color at www.ajtmh.org.

These results are sensitive to changes in the cost of the intervention as well as to the probability of effectiveness among the compared strategies (Figure 1). Results of the probabilistic sensitivity analysis are shown in Figure 2. We note that both alternatives generate similar effectiveness, but the *Aedes*-free intervention is associated with increasing costs.

**Figure 3.**
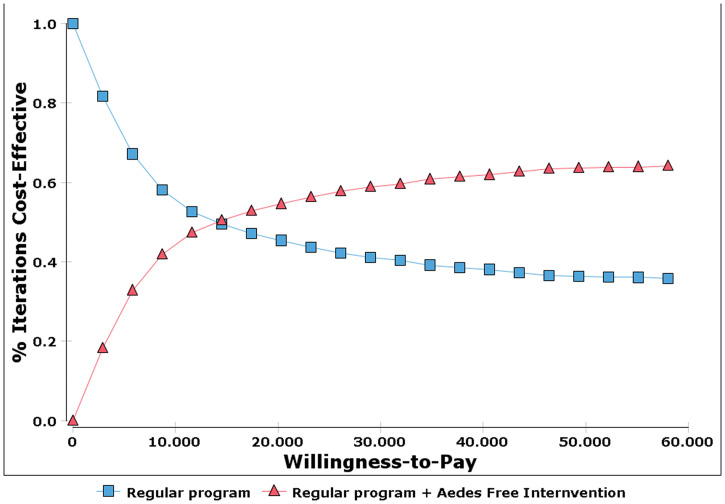
Cost-effectiveness acceptability curve. This figure appears in color at www.ajtmh.org.

Figure 3 shows the cost-effectiveness acceptability curve. As the municipality’s willingness to pay increases, the probability of the *Aedes*-free intervention to be cost-effective also increases. Furthermore, results show that from a threshold of USD12,000 onward, the *Aedes*-free program has a greater probability of being cost-effective compared with the regular program.

In the sensitivity analysis using DALYs as the outcome measure, without intervention, 109 DALYs would be lost in Girardot in 1 year as a result of dengue. With the intervention, this value would reduce to 88 DALYs.

## DISCUSSION

Dengue is one of the main public health problems in Colombia. The high burden of the disease and the significant cost of control programs (e.g., the costs of the prevention and control program in Colombia for the 2010 epidemic year and the 2012 non-epidemic year were 46% and 64% of the total cost of dengue in each year, respectively) reinforce the need for effective strategies for dengue control. If, in addition, such strategies are shown to be cost-effective, resources can be allocated to these interventions and thus contribute to social welfare.

There are few economic evaluations that compare regular programs for the prevention and control of dengue with innovative, multisector, community participation interventions. A recent study conducted in Sri Lanka evaluated dengue vector control interventions in terms of effectiveness and cost-effectiveness and found a positive effect if the interventions are implemented and well-coordinated rigorously across sectors, which could significantly reduce the disease and economic burdens of dengue in endemic settings.^9^

A 5-year intervention consisting of an environmental management strategy with community participation combined with the regular control program was carried out in Santiago de Cuba, between 2000 and 2004. The cost-effectiveness of this program was compared with the regular program.^28^ Both the costs per inhabitant and the entomological indicators (housing index) were comparable before the intervention (2000). The cost-effectiveness ratio for the intervention at 5 years was USD831.1 per focus in the intervention area versus USD2,465.6 in the control area. The intervention generated savings and health benefits that were maintained throughout the observation period.

In our analysis, we found that the *Aedes*-free intervention is potentially cost-effective in Girardot and, given the similar characteristics of this town to other endemic areas in Colombia, we anticipate this strategy could also be implemented in other Colombian areas. One limitation for this national implementation is that the cover needs to be manufactured individually for each house, because the water storage tanks are not of uniform size, which makes them different and production cannot be done for a series of covers.

Measurement of indirect costs is a challenge in most cost-effectiveness analyses, particularly in public health interventions. We included an estimation of indirect costs and therefore contribute to the knowledge in an underrepresented area of research. However, there are several limitations with this analysis. First, the cost-effectiveness threshold on interventions in which the outcome is not measured in terms of quality-adjusted life years is undoubtedly a controversial topic. For this reason, this evaluation considered the expenses that the municipality and inhabitants of Girardot assumed throughout the year. Opening this discussion is of importance for all public health interventions that are not measured in quality-adjusted life years.

Second, when evaluating the effect of the intervention, dengue transmission was reduced significantly in Girardot in 2017, so the number of cases (incidence) in both the intervention and control areas was small, leading to low effectiveness (difference between intervention area and control).

Third, the cost in the intervention area was the cost of the mesh covers plus the cost of the regular program. If a longer term evaluation is made, not all the actions of the regular program have to be carried out, and therefore the sum of intervention costs plus the control program would be less than those that we calculated. This is an interesting area for future research.

Fourth, cost-effectiveness analysis of public health interventions should adopt a long-term time horizon to reflect the fact that many programs only generate population benefits in the long term. However, some public health programs might create short-term benefits, such as the intervention we assessed in this analysis. A 1-year time horizon in the cost-effectiveness analysis provides relevant information to decisionmakers.

In conclusion, a dengue prevention program based on community participation and the implementation of covers impregnated with long-lasting insecticide in water tanks (the most productive dengue vector containers) is a potentially cost-effective option in areas with a high prevalence of dengue. However, given the short follow-up, the results of this analysis should be complemented by local budget-impact analyses to estimate the total cost of implementing the proposed intervention. In addition, local authorities that decide to carry out this policy should ensure coverage of high-risk transmission areas (public spaces such as schools and commercial sites) and provision of an improved surveillance system to maximize the effect of the intervention.
